# Suppression of G6PD induces the expression and bisecting GlcNAc-branched N-glycosylation of E-Cadherin to block epithelial-mesenchymal transition and lymphatic metastasis

**DOI:** 10.1038/s41416-020-1007-3

**Published:** 2020-07-28

**Authors:** Yifei Wang, Qingxiang Li, Lixuan Niu, Le Xu, Yuxing Guo, Lin Wang, Chuanbin Guo

**Affiliations:** 1 Department of Oral and Maxillofacial Surgery, Peking University School and Hospital of Stomatology, 100081 Beijing, China; 2 The Fourth Outpatient Department, Peking University School and Hospital of Stomatology, 100025 Beijing, China

**Keywords:** Oral cancer, Metastasis, Cadherins, Glycosylation, Cancer metabolism

## Abstract

**Background:**

As the rate-limit enzyme of the pentose phosphate pathway, glucose-6-phosphate dehydrogenase (G6PD) plays important roles in tumour progression, but the exact mechanism through which G6PD controls cancer metastasis remains unclear.

**Methods:**

G6PD expression in resected oral squamous cell carcinoma (OSCC) samples was analysed by immunohistochemistry. The effects and mechanism of G6PD suppression on OSCC cell lines were measured by transwell assay, wound healing assay, western and lectin blot, mass spectrometer analysis, ChIP-PCR, and luciferase reporter assay. BALB/c-nude mice were used to establish orthotopic xenograft model.

**Results:**

G6PD expression in the tumours of 105 OSCC patients was associated with lymphatic metastasis and prognosis. In vitro cellular study suggested that G6PD suppression impaired cell migration, invasion, and epithelial-mesenchymal transition. Furtherly, G6PD knockdown activated the JNK pathway, which then blocked the AKT/GSK-3β/Snail axis to induce E-Cadherin expression and transcriptionally regulated *MGAT3* expression to promote bisecting GlcNAc-branched N-glycosylation of E-Cadherin. An orthotopic xenograft model further confirmed that dehydroepiandrosterone reduced lymphatic metastatic rate of OSCC, which was partially reversed by JNK inhibition.

**Conclusions:**

Suppression of G6PD promoted the expression and bisecting GlcNAc-branched N-glycosylation of E-Cadherin via activating the JNK pathway, which thus acted on OSCC metastasis.

## Background

Cancer metastasis is one of the most important factors that contribute to a poor prognosis. To date, accumulating evidences have indicated that glucose metabolism might affect the occurrence of tumour metastasis,^1–3^ which in turn suggests that regulating tumour cell metabolism to prevent tumour metastasis might be a more direct and efficient approach.

The glucose metabolism of tumour cells is much higher than that of normal cells, and it is thought to be an adaptive process to meet the needs of self-proliferation and migration. The pentose phosphate pathway (PPP) is one of the glucose catabolism pathways that is critical for regulating cell growth.^4,5^ Glucose-6-phosphate dehydrogenase (G6PD) is the rate-limiting enzyme in the PPP. The lack of G6PD reduces the generation of nicotinamide adenine dinucleotide phosphate (NADPH), thus causing reactive oxygen species (ROS) accumulation.^6,7^ Studies have demonstrated that the ROS-mediated activation of the c-Jun N-terminal kinase (JNK) pathway impedes tumour progression by inducing cell apoptosis.^8,9^

Epithelial-mesenchymal transition (EMT), the initial step in cancer metastasis, is a process by which epithelial cells lose cell polarity and intercellular connections and gain enhanced cell migration and invasion capacities.^10,11^ E-Cadherin, an EMT regulatory molecule, plays an inhibitory role in cancer metastasis by blocking cell migration or invasion.^12–14^ Moreover, post-translational modification of E-Cadherin, especially bisecting and β1,6-GlcNAc-branched N-glycosylation, plays crucial role in metastasis regulation.^15,16^

In the head and neck region, oral squamous cell carcinoma (OSCC) is the most common epithelial malignancy.^17,18^ Cervical lymphatic metastasis is one of the most important factors contributing to the long-term survival of patients with OSCC.^19^ A salvage operation that includes radical neck dissection and tumour resection is always required for patients with cervical metastasis, and the quality of life of these patients markedly decreases post-surgery.^20^ Hence, there is urgent need for new therapy strategy for patients with metastasis.

An orthotopic xenograft model was performed in BALB/c-nude mice. The OSCC cell line was injected into the tongue. As previous studies reported,^21,22^ the orthotopic injected cells could transfer to cervical lymph nodes, which simulates the lymphatic metastasis of OSCC to some extent.

In this study, we investigated the relationship between glucose metabolism and metastasis in OSCC. Specifically, we examined the role G6PD plays in E-Cadherin expression and post-translational modification. We aimed to evaluate the potential use of G6PD as a diagnostic or therapeutic target for solid tumours.

## Methods

### Patients

Patients diagnosed with OSCC and who underwent surgical resection at the Peking University School and Hospital of Stomatology between 2014 and 2016 were enrolled in the current study. Patients with diabetes, hyperthyroidism, and other metabolic diseases were excluded. Informed consent was obtained from all patients. This study was approved by the Ethics Committee of Peking University School and Hospital of Stomatology (NO. PKUSSIRB-201944044). Telephone follow-up was performed to analyse the survival trends among these patients.

### Immunohistochemistry (IHC)

The tissue sections were collected, deparaffinised, and rehydrated in xylene and a gradient ethyl alcohol series. Antigen retrieval was performed in 0.01 M sodium citrate buffer (pH = 6.0) in a pressure cooker for 3 min. After they cooled naturally to room temperature (RT, ~25 °C), the samples were treated with 3% (w/v) hydrogen peroxide for 10 min at RT and incubated with anti-G6PD antibody (1:1000, Abcam, Cambridge, MA, USA) overnight at 4 °C. Then, the samples were stained with horseradish peroxidase-labelled secondary antibody (Zhongshan Biosciences Inc., Beijing, China) and haematoxylin. All images were analysed with an optimal microscope (Olympus).

### Cell culture

The human OSCC cell lines CAL27 (RRID: CVCL_1107) and WSU-HN6 (RRID: CVCL_5516) were used in this study. CAL27 was obtained from American Type Culture Collection (ATCC, CRL-2095) and WSU-HN6 was obtained from Central Laboratory of Peking University School and Hospital of Stomatology. The cells were authenticated by STR analysis and mycoplasma detection before used. Both cells were cryopreserved for more than 6 months and the general length of time between thawing and use was not exceeding 3 months. The cells were cultured in DMEM (Life technology, Carlsbad, CA, USA) containing 10% FBS (Gibco, Waltham, MA, USA) and 1% penicillin/streptomycin (Gibco) in a humidified atmosphere containing 5% CO_2_/95% air at 37 °C.

### siRNA transfection and drug treatment

When the cells reached 50–60% confluence, the siRNA duplexes, siNC or siG6PD (Ruibo Biosciences Inc., Guangzhou, China), were transfected using the JetPrimer regent (Polyplus transfection, Strasbourg, France). The siRNA (20 μM) and JetPrimer regent (1:1) were dissolved in transfection buffer at a ratio of 1:50. After 10-minute incubation at RT, the complex was added to the DMEM at a ratio of 1:10. The final concentration of siRNA was 40 nM. After 8-h incubation, the transfection medium was washed out and serum-free DMEM was added to inhibit cell proliferation. For the following functional assays, siRNA transfection was performed 24 h before each assay, accompanied with western blot for evaluating transfection effect.

For drug administration, dehydroepiandrosterone (DHEA, 50 μM, Selleck, Houston, TX, USA) was added to the experimental medium when the cells reached 70–80% confluence. For the rescue assays, SP600125 (10 μM, Selleck) was added 12 h before siRNA transfection, while SC79 (8 μM, Selleck) was added 12 h after siRNA transfection.

### Wound healing assay

After treated with siRNA or drugs, the cells were scratched with pipette tips, and the incubation in serum-free DMEM continued. Images were taken every 12 h with an optimal microscope (Olympus) and analysed using ImageJ (NIH, USA).

### Transwell assay

After treated with siRNA or drugs, the cells were resuspended in serum-free DMEM at a concentration of 10^5^ cells per well. They were then transferred to the transwell chambers (Millipore, Darmstadt, Germany) in a 24-well plate containing DMEM with 20% serum. WSU-HN6 cells were harvested after 8-h incubation, while CAL27 cells were harvested after 24 h, according to invasiveness of the different cells. The chambers were then stained with 0.1% crystal violet (Solarbio, Beijing, CN) for 30 min at RT. The images were obtained with an optimal microscope (Olympus) and analysed using ImageJ.

### Immunofluorescence

After treated with siRNA or drugs, the cells were fixed in 4% paraformaldehyde for 15 min at RT, followed by incubation in 0.1% (v/v) Triton-100-PBS. Subsequently, the cells were blocked with 10% goat serum (Zhongshan Biosciences Inc.) and then incubated with anti-cortactin antibody (1:1000, Abcam) overnight at 4 °C. The cells were then stained with FITC-labelled secondary antibody (Zhongshan Biosciences Inc.) for 1 h at RT, following incubation with phalloidin-iFluor 594 (1:1000, Abcam) for 30 min at RT. Nuclear staining was performed by incubation with 4ʹ,6-diamidino-2-phenylindole (DAPI, Zhongshan Biosciences Inc.). The images were then captured using a fluorescent optimal microscope (Olympus).

### Western blot

Total proteins were extracted from the cells using RIPA buffer (Huaxingbio, Beijing, China) containing a protease inhibitor and phosphatase inhibitors. A total of 100 μg of protein was separated in a 10% (w/v) gel using sodium dodecyl sulphate polyacrylamide gel electrophoresis (SDS-PAGE) and then transferred to PVDF membranes (BioRad, Hercules, CA, USA). Thereafter, the membranes were incubated in Tris buffered saline with 0.1% (v/v) tween-20 (TBST) containing 5% (w/v) non-fat milk for 1 h at RT, followed by incubation with primary antibody (Supplementary Table 1) in 5% (w/v) BSA-TBST overnight on a shaker at 4 °C. The membranes were then stained with the appropriate secondary antibodies. Immunoreactive bands were visualised using an enhanced chemiluminescent (ECL) detection reagent (Cwbiotech, Beijing, China) and quantified by densitometry using Quantity One 4.6 software (BioRad). The relative expression was normalised to that of ribosomal protein S18 (RPS18).

### Lectin blot analysis of immunoprecipitated E-Cadherin

E-Cadherin was separated from cell lysate by immunoprecipitation. The detail procedure was described in Supplementary materials and methods. Thereafter, the immunoprecipitated E-Cadherin were applied to 10% (w/v) SDS-PAGE, and electrophoretically transferred to PVDF membranes. For lectin blots, 1x Carbo Free Blocking Solution (Vector Laboratories, Burlingame, CA, USA) was used for blocking, followed by biotinylated Phaseolus Vulgaris Erythroagglutinin (PHA-E, 1:500, Vector Laboratories) or biotinylated Phaseolus Vulgaris Leucoagglutinin (PHA-L, 1:500, Vector Laboratories) incubation overnight at 4 °C. Then, the membrane was incubated with streptavidin-horseradish peroxidase (Vector Laboratories) for 30 min at RT. Immunoreactive bands were visualised following western blot protocol. The relative expression was normalised to that of immunoprecipitated E-Cadherin.

### Real-time quantitative polymerase chain reaction (qPCR)

Total RNA from WSU-HN6 or CAL27 cells was extracted using TRIzol reagent (Ambion, Waltham, MA, USA) according to the product’s protocol. The RNA quantity and purity were measured with a spectrophotometer (Bio Tek). The cDNA was synthesised using a reverse transcription kit (Promega, Madison, WI, USA). Real-time PCR assays were performed using SYBR Green (Roche, Switzerland) in the ABI 7500 Real-Time PCR Detection System (Applied Biosystems). Ribosomal protein S18 (RPS18) was used as the endogenous standard. The PCR programme consisted of pre-denaturation at 95 °C for 10 min followed by 40 cycles amplification of 95 °C for 15 s and 60 °C for 1 min. The primer was synthesised by Shanghai Shenggong Co., Ltd., and its sequence is provided in Supplementary Table 2. The relative expression level was normalised to the amount of RPS18 and calculated using the 2^−ΔΔCt^ method.

### Chromatin immunoprecipitation assay

Chromatin immunoprecipitation assay (ChIP) assays were performed using the EzChIP Assay Kit (Millipore) according to the manufacturer’s instructions. Briefly, cells were cross-linked with 37% formaldehyde, pelleted, and resuspended in lysis buffer. The cells were then sonicated and centrifuged to remove the insoluble material. Consequently, the supernatants were collected and incubated overnight with anti-c-Jun antibodies (1:200, CST, Danvers, MA, USA) and Protein G agarose beads. The beads were washed, and the precipitated chromatin complexes were collected, purified, and de-crosslinked at 62 °C for 5 h, followed by DNA purification and qPCR detection.

### Luciferase reporter assay

Luciferase reporter assays were performed according to the manufacturer’s instructions (Promega). Briefly, cells were seeded in 24-well plates and treated with siRNA or inhibitors. Then, cells were transfected with 1 μg of promoter luciferase reporter or empty vector. Twenty-four hours after transfection, the cells were harvested in 1x lysis buffer and subjected to a single freeze-thaw cycle to ensure complete lysis. Cell lysates were transferred to the microcentrifuge tubes, vortexed for 3 min, and then centrifuged at 12,000×*g* for 5 min at 4 °C. Ten microliters of the supernatant were mixed with 10 μl of the Luciferase Assay Reagent per tube and measured using a luminometer to determine the luciferase activity.

### Orthotopic xenograft studies

Eighteen 4-week-old female BALB/c-nude mice (Vital River Laboratory Animal Technology, Beijing, China) were housed in a specific pathogen free environment with temperature of 22 ± 1 °C, relative humidity of 50 ± 1% and a light/dark cycle of 12/12 h. All animal studies (including the mouse euthanasia procedure) were performed in compliance with the regulations and the Peking University institutional animal care guidelines and conducted according to the AAALAC and the IACUC guidelines (NO. LA2019011).

The mice were randomly divided into three groups: Control, Treatment, and Rescue (*n* = 6 for each group) and were anesthetised with 2% pentobarbital sodium (50 mg/kg weight). The orthotopic OSCC mouse model was established according to a previously described approach.^21,22^ After 6 day, vector (2% DMSO, 30% PEG300, and 5% Tween-80 diluted in sterilised distilled water), DHEA (80 mg/kg weight), or DHEA and SP600125 (15 mg/kg weight) were intraperitoneally injected into each mouse every 3 day. After 20 day, the mice were euthanised by cervical dislocation. The lymph nodes were processed for histology and immunohistochemistry to detect metastasis. Three mice from each group were randomly selected, and their tumour tissues were used for western blot, lectin blot and immunofluorescence analysis.

### Statistical analysis

The Mann–Whitney *U* test was used to analyse the relationship between G6PD expression and lymphatic metastasis in patients with OSCC. The Chi-square test was used to analyse the relationship between G6PD expression and the clinical parameters of the patients. The Kaplan–Meier method was used for survival analysis. T-tests were used to compare the difference between two groups, including the results of the wound healing assay, transwell, western blot, qPCR, luciferase reporter assays, and mass spectrometer analysis. A *P* value of <0.05 was considered significantly different. In addition, all statistical analyses were performed using SPSS 22.0 for Windows. Results are expressed as mean ± SEM.

## Results

### High expression of G6PD was associated with high lymphatic metastasis rate and poor prognosis in patients with OSCC

**Fig. 1:**
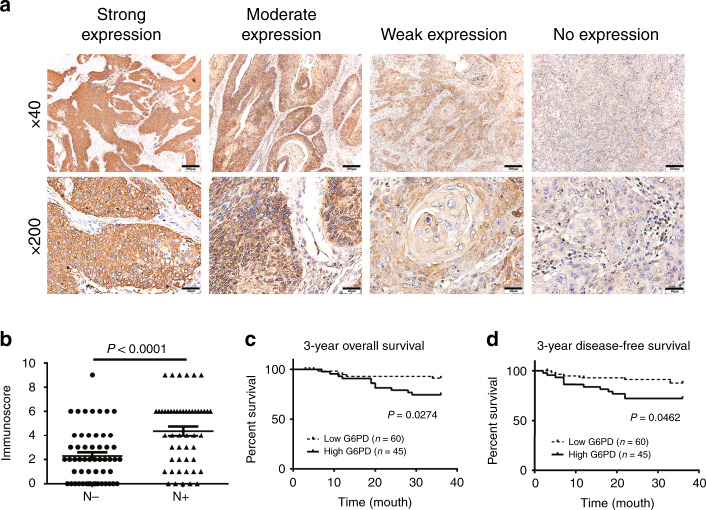
Expression of G6PD was associated with high metastatic rate and poor prognosis of OSCC. **a** The expression pattern of G6PD in OSCC samples. b The relationship between G6PD expression and lymphatic metastasis of OSCC was analysed by using the Mann–Whitney U test the evaluate G6PD expression. “N−” indicates no metastasis (*n* = 55), while “N+” indicates the presence of metastasis (*n* = 50). **c**, **d** Three-year overall survival rate (**c**) and 3-year disease-free survival rate (**d**) analysed using the Kaplan–Meier Method and Log rank test.

To explore the association between the expression of G6PD and the clinical characters of OSCC, 105 OSCC samples were collected and analysed by immunochemical methods. Different expression patterns of G6PD (Fig. 1a) were evaluated using the immunoscore method.^23^ The results indicated that the expression of G6PD was significantly associated with the pathological grade, clinical stage, T stage, and lymphatic metastasis (Supplementary Table 3). Among these, G6PD expression and lymphatic metastasis had the strongest correlation, which was further confirmed by a non-parameters Wilcoxon symbols test (Fig. 1b). In addition, a lower 3-year overall and disease-free survival rate were observed in patients with high expression of G6PD than in those with low G6PD expression (Fig. 1c, d).

### Suppression of G6PD inhibited cell migration, invasion, and EMT of OSCC

**Fig. 2:**
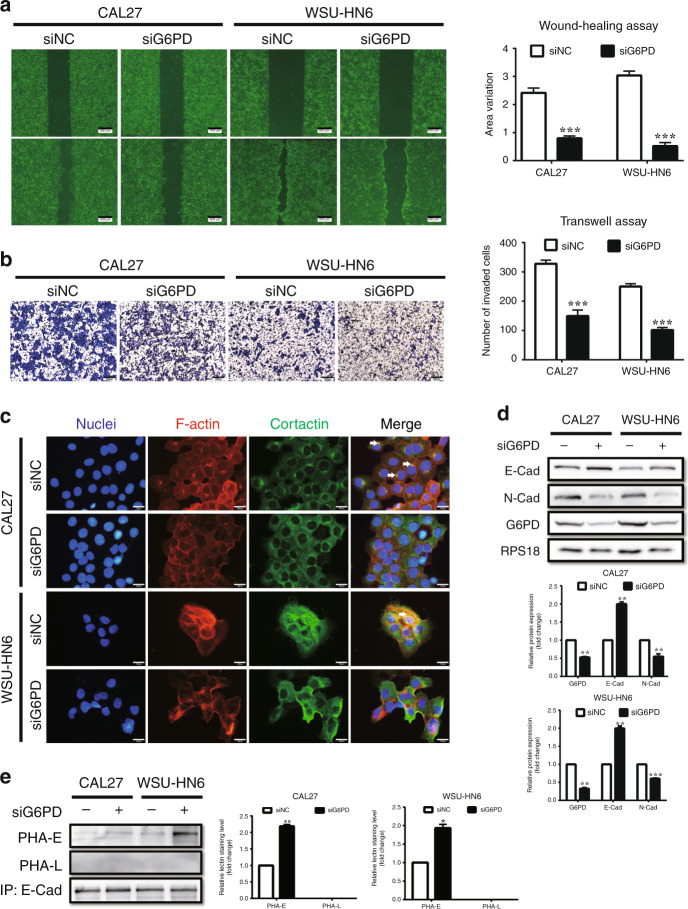
Knockdown of G6PD inhibits migration, invasion, and EMT of OSCC cell lines. **a** Wound healing assays in CAL27 and WSU-HN6 cells treated with siNC or siG6PD. Five different fields were recorded and measured. **b** Transwell assays in CAL27 and WSU-HN6 cells treated with siNC or siG6PD. Five different fields were recorded and measured. **c** Co-localisation of F-actin and cortactin was evaluated using phalloidin and immunofluorescence staining to reveal invadopodia of the cells. F-actin was labelled by red fluorescence, and cortactin was labelled by green fluorescence. White arrows indicate the co-localisation of F-actin and cortactin. **d** Western blot analysis of E-Cadherin, N-Cadherin and G6PD in CAL27 and WSU-HN6 cells treated with siNC or siG6PD. Densitometric analysis of protein expression relative to RPS18 levels (*n* = 2 per group). **e** Lectin blot analysis of PHA-E and PHA-L in CAL27 and WSU-HN6 cells treated with siNC or siG6PD. Densitometric analysis of protein expression relative to E-Cadherin levels (*n* = 2 per group). The data in the graph are presented as the mean ± SEM. *P < 0.05, **P < 0.01, ***P < 0.001.

To explore the effect of G6PD on tumour metastasis, we firstly investigated whether G6PD suppression was associated with cell migration and invasion. G6PD suppression mediated by siRNA or inhibitor (DHEA) was assessed in WSU-HN6 and CAL27 cell lines. In the following experiments, ‘siG6PD-1’ was used for G6PD knockdown, and 50 μM DHEA was used for suppressing G6PD activity (Supplementary Fig. 1A–D). The wound healing assay results showed that cells treated with siG6PD or DHEA had slower migration than the control group (Fig. 2a, Supplementary Fig. 1E). The transwell assay results revealed the reduction of invasive ability in these cells compared to that in the control group (Fig. 2b, Supplementary Fig. 1F).

As the important role of EMT plays in cancer metastasis, we paid attention to this process. Cells that undergo EMT always manifest morphology changes and reorganise their cortical actin cytoskeleton into one that enables dynamic cell elongation and directional motility.^14^ We found that the cells treated with siG6PD appeared to form fewer branches and had a more regular form than control (Supplementary Fig. 2A). Immunofluorescent detection of F-actin and cortactin revealed less co-localisation after G6PD knockdown (Fig. 2c). Western blot analysis also indicated the downregulation of the mesenchymal marker N-Cadherin and the activation of the epithelial marker E-Cadherin (Fig. 2d). Furthermore, as bisecting and β1,6-GlcNAc-branched N-glycosylation of E-Cadherin is the most important functional post-translation modification associated with EMT,^15^ we detected them on immunoprecipitated E-Cadherin. Lectin blot analysis revealed that bisecting GlcNAc-branched N-glycosylation of E-Cadherin obviously increased after G6PD knockdown, while the level of β1,6-GlcNAc-branched N-glycosylation of E-Cadherin was fairly low in the two OSCC cell lines (Fig. 2e). Through mass spectrometer analysis in WSU-HN6 cell line, we found G6PD knockdown resulted in enhanced intensity of N-glycans with bisecting GlcNAc-branched structure on E-Cadherin, while led to less effect on β1,6-GlcNAc-branched N-glycans (Supplementary Table 4 and Supplementary Fig. 2B, C). These results represented the reversal of EMT in OSCC cell lines in response to G6PD knockdown.

### JNK pathway inhibitor reversed the inhibition of cell migration, invasion, and EMT caused by G6PD knockdown

**Fig. 3:**
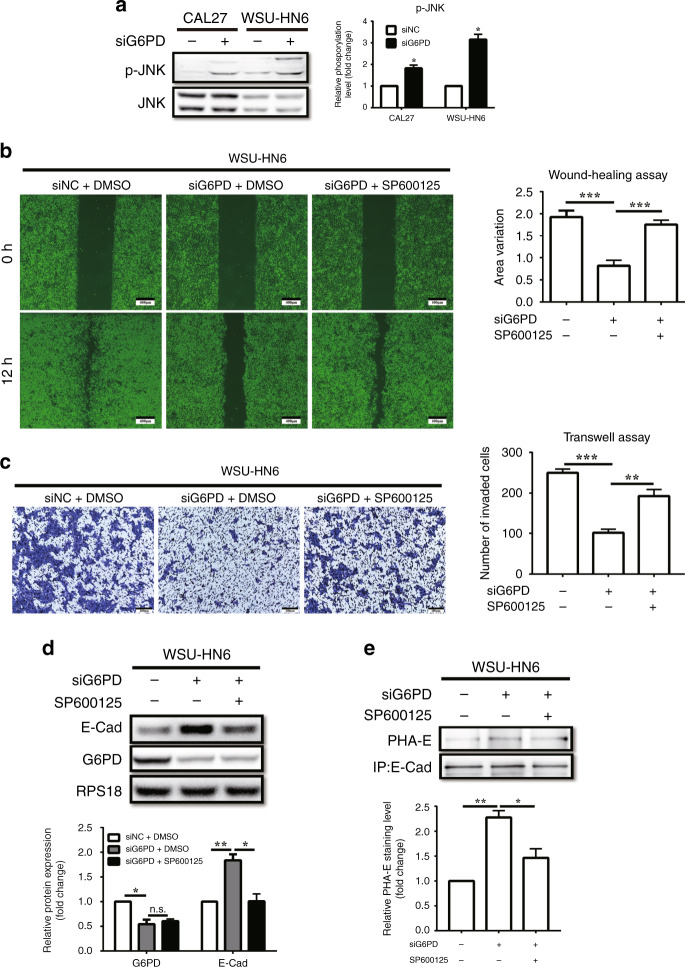
JNK inhibition reversed the G6PD-knockdown-mediated induction of cell migration, invasion, and EMT suppression. **a** Western blot analysis of p-JNK (Thr183/Tyr185) in CAL27 and WSU-HN6 cells treated with siNC or siG6PD. Densitometric analysis of protein phosphorylation relative to JNK levels (*n* = 2 per group). **b** Wound healing assays in WSU-HN6 cells treated with siNC, siG6PD, or siG6PD with SP600125 (10 μM). Five different fields were recorded and measured. **c** Transwell assays in WSU-HN6 cells treated with siNC, siG6PD, or siG6PD with SP600125 (10 μM). Five different fields were recorded and measured. **d** Western blot analysis of E-Cadherin and G6PD in WSU-HN6 cells treated with siNC, siG6PD, or siG6PD with SP600125 (10 μM). Densitometric analysis of protein expression relative to RPS18 levels (*n* = 2 per group). **e** Lectin blot analysis of PHA-E in WSU-HN6 cells treated with siNC, siG6PD, or siG6PD with SP600125 (10 μM). Densitometric analysis of protein expression relative to E-Cadherin levels (*n* = 2 per group). The data in the graph are presented as the mean ± SEM. *P < 0.05, **P < 0.01, ***P < 0.001, n.s.: *P* > 0.05.

It is known that G6PD knockdown impairs NADPH production and causes ROS accumulation, which then promotes the activation of JNK and tumour regression through DNA damage repair.^24^ Herein, the metabolites belong to PPP were firstly analysed by mass spectrum to confirm that G6PD knockdown decreased the level of NADPH in both OSCC cell lines (Supplementary Fig. 3A, B), accompany with the increase of ROS levels (Supplementary Fig. 3C). Besides that, the JNK pathway was also activated as expected (Fig. 3a). Thereafter, we wandered whether the activated JNK pathway mediated by G6PD-knockdown participates in the effects on cell migration, invasion, and EMT, thus the JNK inhibitor SP600125 was used to clarify the interactions. The wound healing assay results showed that it reversed the inhibition of cell migration induced by G6PD knockdown (Fig. 3b, Supplementary Fig. 3D). The transwell assay revealed that the impairment of cell invasion capacity caused by G6PD knockdown could also be rescued by SP600125 (Fig. 3c, Supplementary Fig. 3E). Moreover, western blot analysis showed that the effect on the expression of E-Cadherin following G6PD knockdown was reversed by SP600125, while the expression of G6PD did not change (Fig. 3d, Supplementary Fig. 3F). In addition, as bisecting GlcNAc-branched N-glycosylation of E-Cadherin obviously increased after G6PD knockdown (Fig. 2e), we also paid attention on it. The lectin blot further confirmed the regulatory effect of JNK signalling on the G6PD-knockdown-induced bisecting GlcNAc-branched N-glycosylation of E-Cadherin (Fig. 3e, Supplementary Fig. 3G).

### JNK inactivation reversed the G6PD-knockdown-induced expression of E-Cadherin via the AKT/GSK-3β/Snail axis

**Fig. 4:**
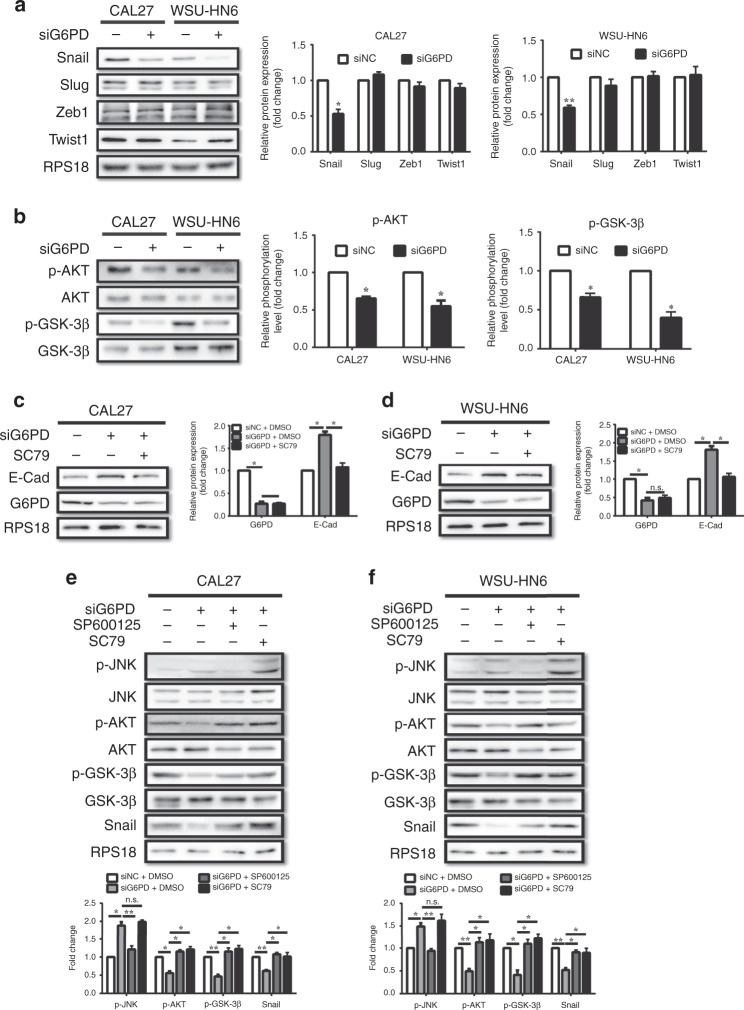
Knockdown of G6PD promoted E-Cadherin expression via the JNK/AKT/Snail axis. **a** Western blot analysis of Snail, Slug, Zeb1 and Twist1 in CAL27 and WSU-HN6 cells treated with siNC or siG6PD. Densitometric analysis of protein expression relative to RPS18 levels (*n* = 2 per group). **b** Western blot analysis of p-AKT (Ser473) and p-GSK-3β (Ser9) in CAL27 and WSU-HN6 cells treated with siNC or siG6PD. Densitometric analysis of protein phosphorylation relative to AKT or GSK-3β levels (*n* = 2 per group). **c**, **d** Western blot analysis of E-Cadherin and G6PD in CAL27 (**c**) and WSU-HN6 **(d)** cells treated with siNC, siG6PD, or siG6PD with SC79 (8 μM). Densitometric analysis of protein expression relative to RPS18 levels (*n* = 2 per group). **e**, **f** Western blot analysis of p-JNK (Thr183/Tyr185), p-AKT (Ser473), p-GSK-3β (Ser9), and Snail in CAL27 (**e**) and WSU-HN6 (**f**) cells treated with siNC, siG6PD, siG6PD with SP600125 (10 μM), or siG6PD with SC79 (8 μM). Densitometric analysis of protein expression and phosphorylation relative to JNK, AKT, GSK-3β, or RPS18 levels (*n* = 2 per group). The data in the graph are presented as the mean ± SEM. *P < 0.05, **P < 0.01, ***P < 0.001, n.s.: *P* > 0.05.

To further investigate how the JNK pathway participates in the G6PD-knockdown-induced reversion of EMT, the expression levels of transcription factors associated with EMT, including Snail, Slug, Twist1, and Zeb1, were analysed. Interestingly, only Snail was significantly downregulated after G6PD knockdown in both OSCC cell lines (Fig. 4a). Snail, an important transcription factor in embryonic development and cancer progression, is controlled by the AKT/GSK-3β pathway,^25,26^ which was also inactivated after the knockdown of G6PD (Fig. 4b). Moreover, SC79, an agonist of AKT, was used to clarify the interaction between AKT and the G6PD-knockdown activation of the JNK pathway. We found that SC79 could also reverse the G6PD-knockdown-induced increase in E-Cadherin expression (Fig. 4c, d), as well as the inhibition of cell migration and invasion capacity (Supplementary Fig. 4A–D). Besides that, JNK inhibition reversed the G6PD-knockdown-induced replenishment of phosphorylated AKT and GSK-3β and the expression of Snail, while AKT reactivation had no significant effect on the phosphorylation of JNK (Fig. 4e, f).

### JNK inactivation reversed the G6PD-knockdown-mediated induction of bisecting GlcNAc-branched N-glycosylation of E-Cadherin by transcriptionally regulating *MGAT3*

**Fig. 5:**
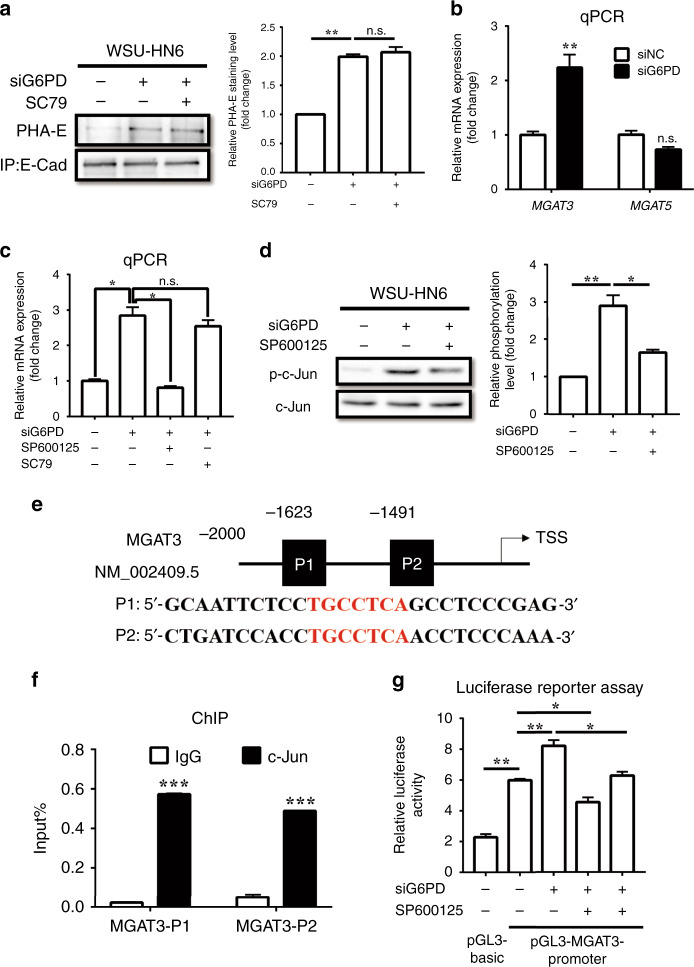
Knockdown of G6PD promoted bisecting GlcNAc-branched N-glycosylation of E-Cadherin by transcriptionally regulating the expression of *MGAT3*. **a** Lectin blot analysis of PHA-E on immunoprecipitated E-Cadherin in WSU-HN6 cells treated with siNC, siG6PD, or siG6PD with SC79 (8 μM). Densitometric analysis of protein expression relative to E-Cadherin levels (*n* = 2 per group). **b** qPCR of *MGAT3*, and *MGAT5* in WSU-HN6 cells treated with siNC or siG6PD. mRNA expression relative to RPS18 levels (*n* = 2 per group). **c** qPCR of *MGAT3* in WSU-HN6 cells treated with siNC, siG6PD, siG6PD with SP600125 (10 μM), or siG6PD with SC79 (8 μM). mRNA expression relative to RPS18 levels (*n* = 2 per group). **d** Western blot analysis of p-c-Jun (Ser73) in WSU-HN6 cells treated with siNC, siG6PD, or siG6PD with SP600125 (10 μM). Densitometric analysis of protein phosphorylation relative to c-Jun levels (*n* = 2 per group). **e** The putative c-Jun binding site (Red characters) of the *MGAT3* promoter was predicted using the JASPAR database. **f** ChIP assay of c-Jun and the *MGAT3* promoter in the WSU-HN6 cell line (*n* = 3 per group). **g** Luciferase reporter assay of *MGAT3* promoter function in WSU-HN6 cells treated with siNC, siG6PD, SP600125, or siG6PD with SP600125 (10 μM) (*n* = 3 per group). The data in the graph are presented as the mean ± SEM. *P < 0.05, **P < 0.01, n.s.: *P* > 0.05.

Except the above mechanism on E-Cadherin expression by JNK pathway in G6PD-knockdown, we further explored the mechanism by which G6PD induced the bisecting GlcNAc-branched N-glycosylation of E-Cadherin. Unexpectedly, SC79 could not reverse the increase in the bisecting GlcNAc-branched N-glycosylation of E-Cadherin (Fig. 5a, Supplementary Fig. 5A). However, *MGAT3*, which encodes acetylglucosamine transferase (GnT) III, the enzyme that catalyses the generation of bisecting GlcNAc was significantly upregulated after G6PD knockdown, while the expression of *MGAT5*, which encodes GnT-V, thus catalyses β1,6-GlcNAc, revealed none of obviously variation (Fig. 5b, Supplementary Fig. 5B). By detecting the level of total bisecting GlcNAc and β1,6-GlcNAc of total proteins, we found G6PD knockdown resulted in enhanced bisecting GlcNAc while decreased β1,6-GlcNAc, which indicated that the function of *MGAT3* (or GnT-III) was enhanced by G6PD knockdown (Supplementary Fig. 5C). Further investigation of the expression of *MGAT3* (and GnT-III) revealed that SP600125 could reverse G6PD-knockdown-induced *MGAT3* expression, while SC79 could not (Fig. 5c, Supplementary Fig. 5D–F). These results indicated that JNK pathway participated in G6PD-knockdown-induced bisecting GlcNAc-branched N-glycosylation of E-Cadherin via another approach, instead of AKT pathway.

Therefore, we searched the GeneCards and JASPAR database for putative transcription factors of *MGAT3*. Interestingly, we found that c-Jun was the putative transcription factor for *MGAT3*. The phosphorylation of c-Jun increased after G6PD knockdown, and this effect was reversed by JNK inhibition (Fig. 5d, Supplementary Fig. 6A). Furthermore, we searched 2 kb of the promoter regions of *MGAT3* and found two putative c-Jun binding sites (TGCCTCA region) (Fig. 5e). Luciferase reporter assays demonstrated that the mutation of these binding sites reduced *MGAT3* promoter reporter activity (Supplementary Fig. 6B–D). Chromatin immunoprecipitation (ChIP) assays revealed that endogenous c-Jun was recruited to the regions containing the binding sites (Fig. 5f, Supplementary Fig. 6E). Moreover, siG6PD increased the reporter activity of *MGAT3* promoters, while inhibition of c-Jun by SP600125 decreased the reporter activity (Fig. 5g, Supplementary Fig. 6F).

### Suppression of G6PD inhibited OSCC lymphatic metastasis by activating the JNK pathway in orthotopic xenograft model

**Fig. 6:**
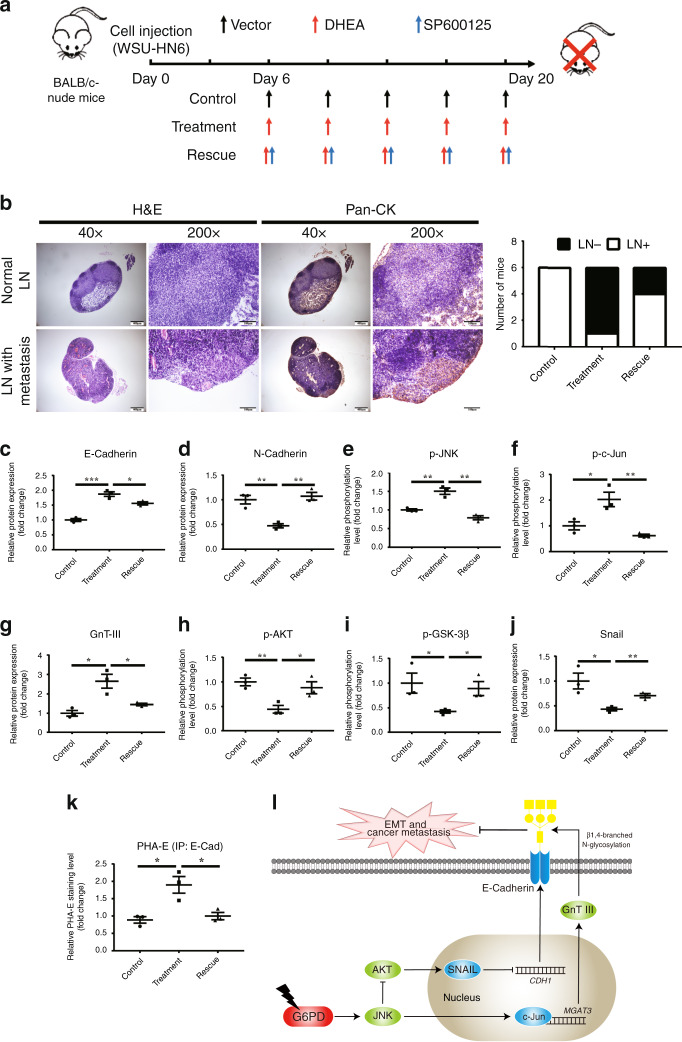
Suppression of G6PD by DHEA inhibited lymphatic metastasis in an orthodontic xenograft animal model via activation of the JNK pathway. **a** The scheme of the animal experiment. **b** Representative HE and immunohistochemical staining photos of pan-CK in the lymph nodes with or without metastasis. The numbers of mice with or without metastasis in each group (*n* = 6) are indicated in the histogram. **c–j** Western blot of E-Cadherin, N-Cadherin, p-JNK, p-c-Jun, GnT-III, p-AKT, p-GSK-3β, and Snail in tumour tissue. Densitometric analysis of protein expression or phosphorylation relative to RPS18, JNK, c-Jun, AKT, or GSK-3β levels. **k** Lectin blot analysis of PHA-E on immunoprecipitated E-Cadherin in tumour tissue. Densitometric analysis of protein expression relative to E-Cadherin levels. **l** The diagram for explaining the mechanism that how G6PD knockdown promoted the expression and bisecting GlcNAc-branched *N*-glycosylation of E-Cadherin. The data in the graph are presented as the mean ± SEM. *P < 0.05, **P < 0.01, ***P < 0.001.

To further confirm the role of JNK pathway in G6PD-suppression-induced OSCC metastasis inhibition, WSU-HN6 cells were injected into the tongue of BALB/c-nude mice to simulate the cervical metastasis (Fig. 6a). G6PD activity was reduced by DHEA treatment, which could not be reversed by SP600125 (Supplementary Fig. 7A), and the weight of mice showed no significant difference among groups (Supplementary Fig. 7B). As expected, all mice injected with the vector developed cervical lymphatic metastasis, while only one out of six mice treated with DHEA developed metastasis. The mice injected with DHEA and SP600125 had an increased metastatic rate of 66.67% (4/6) (Fig. 6b). Furthermore, ex vivo analysis of dissected tumour samples revealed that the expression of E-Cadherin and GnT-III, in addition with the phosphorylation of JNK and c-Jun was enhanced, while the phosphorylation of AKT and GSK-3β and the expression of N-Cadherin and Snail were reduced (Fig. 6c–j, Supplementary Fig. 7C). Lectin fluoresce also revealed that the expression of E-Cadherin and total bisecting GlcNAc-branched N-glycosylation of tumour tissues were enhanced by DHEA treatment (Supplementary Fig. 7D), and the bisecting GlcNAc-branched N-glycosylation of E-Cadherin was enhanced by DHEA treatment (Fig. 6k, Supplementary Fig. 7E). Notably, inhibition of JNK by SP600125 reversed these variances above. These results indicated that JNK pathway has a core role in G6PD-inhibition-induced lymphatic metastasis suppression of OSCC.

## Discussion

Identifying lymphatic metastasis of OSCC is a critical step when preforming the diagnosis and treatment of the disease. Exploring biomarkers for use in metastasis prediction and clarifying the mechanism of lymphatic metastasis are essential for the evaluation and treatment of OSCC. Metabolic reprograming is an important character of cancer.^27^ According to Warburg effect and additional study,^28,29^ the glucose taken by cancer cells always draws into glycolysis and PPP. Metastatic tumours reveal enhanced PPP flux for resistance of microenvironment change.^30^ G6PD, the rate-limited enzyme in the pentose phosphate pathway, is a critical enzyme that regulates cell proliferation and tumour formation.^31,32^ It has been reported that G6PD deficiency reduces the susceptibility for developing endodermal-origin cancer,^33^ but the role and mechanism of G6PD plays in cancer metastasis remains unclear.

In this study, we focused on exploring diagnostic and therapeutic value of G6PD in OSCC. We examined tumour samples obtained from 105 OSCC patients and further explored the role of G6PD in cancer metastasis in vitro and in vivo. We found that its expression is associated with lymphatic metastasis and patients’ prognosis. Suppression of G6PD by siRNA or DHEA inhibited cell migration, invasion and EMT process in vitro, and inhibited lymphatic metastasis in an orthodontic xenograft in mice. Most notably, we demonstrated that G6PD regulated not only the expression of E-Cadherin, but also bisecting GlcNAc-branched N-glycosylation of the molecular (Fig. 6l). To our best knowledge, it is the first time to uncover the regulatory effect of glucose metabolism related molecules on the function of E-Cadherin. The dual modulation of G6PD on E-Cadherin closely associates glucose metabolism with cancer metastasis.

As for the mechanism, we found G6PD knockdown resulted in downregulation of Snail, but not affected the expression of other EMT-associated transcription factors, Slug, Twist1, and Zeb1. According to literature review, the expression of the transcription factors was controlled by different pathways. AKT/GSK-3β is a classical pathway that regulates the expression of Snail, while has less effect on other EMT-associated transcription factors.^25,26^

Moreover, there have been conflicting reports on the role of the ROS-induced JNK pathway in cancer progression.^34^ It was reported that ROS accumulation is a key factor for tumour formation.^35^ However, ROS-induced JNK activation was also found to have an anti-cancer effect via inducing cell apoptosis.^8,36^ Herein, we demonstrated that the G6PD-suppression-induced activation of the JNK pathway impeded the EMT process and lymphatic metastasis of OSCC. These findings shed light on the mechanism for the inhibition of metastasis by ROS accumulation.

Besides that, we also focused on the function of E-Cadherin during EMT and cancer metastasis. Bisecting and β1,6-GlcNAc-branched N-glycosylation are two main functional types of glycosylation in cancer metastasis. The former inhibits tumour metastasis by promoting cell adhesion and impairing the EMT process, while the latter breaks the E-Cadherin/β-catenin complex, thus promoting cell migration and invasion. These two types of N-glycosylation are catalysed by two GnTs, GnT-III and GnT-V, which are encoded by the *MGAT3* and *MGAT5* genes, respectively.^16,37^ In our study, G6PD knockdown induced the expression and function (revealed by the level of total bisecting GlcNAc) of *MGAT3*, in accordance with bisecting GlcNAc-branched N-glycosylation of E-Cadherin. Although β1,6-GlcNAc-branched N-glycosylation of E-Cadherin and the expression of *MGAT5* revealed less variations, the level of total β1,6-GlcNAc was decreased. As it was reported that GnT-III competitively inhibits the activity of GnT-V,^38,39^ we assume that the enhanced expression of GnT-III inhibited the function of GnT-V. These results indicated that G6PD knockdown resulted in enhanced *MGAT3* expression and function to reverse EMT, thus impaired cancer metastasis.

In our study, we also demonstrated that G6PD knockdown decreased the level of NADPH, thus enhanced cellular ROS level and activated JNK pathway. However, the level of another metabolite, ribose-5-phosphate, was decreased in CAL27, while enhanced in WSU-HN6 cell line. We speculated that non-oxidative PPP, which provided a bridge between PPP and glycolysis,^4^ might play a role, especially in WSU-HN6 cell line. Besides, according to the results, glyceraldehyde-3-phosphate (G-3-P), the metabolite connects PPP with glycolysis, was slightly increased in WSU-HN6 while decreased in CAL27 cell line, which provided evidence to support the hypothesis. In general, our results indicated that G6PD knockdown in OSCC cell lines mainly impaired glutathione synthesis but led to unclear effect on nucleotide synthesis.

Meanwhile, the expression and N-glycosylation of E-Cadherin could interact with each other. E-Cadherin could induce the expression of *MGAT3*; while the *MGAT3* knockdown could lead to cytoplasmic accumulation of E-Cadherin.^40^ We considered that Wnt/β-catenin pathway might attend in the interaction between E-Cadherin expression and N-glycosylation. The intracellular part of E-Cadherin can bind with β-catenin and form a complex to maintain β-catenin.^41^ When Wnt/β-catenin is activated, β-catenin accumulates in cytoplasm where it is phosphorylated and transferred into nucleus to regulate transcription. *DPAGT1*, encoding the enzyme catalysed the initial step of N-glycan biosynthesis,^42^ has been reported to be a target of β-catenin.^43^ Its overexpression in oral cancer has been associated with aberrant N-glycosylation and enhanced cell migration.^44,45^ This indicated that E-Cadherin-β-catenin-*DPAGT1* cycle could engage in the crosstalk between the expression and N-glycosylation of E-Cadherin. However, N-glycosylation of protein is a complicated process that needs to be further investigated.

In summary, we demonstrated that G6PD is important for cancer metastasis. Suppression of G6PD blocks EMT of OSCC cell lines and reduces lymphatic metastasis in OSCC orthotopic xenograft model, which indicates that targeting G6PD in cancer cells could be an effective therapeutic strategy. We proposed that G6PD could be potential diagnostic biomarker and therapeutic target for lymphatic metastasis of epithelial malignancies.

## Supplementary information

Supplemental materials

## Data Availability

The data that support the findings of this study are available from the corresponding author upon reasonable request.
