# Supplementary material for: Suppression of G6PD induces the expression and bisecting GlcNAc-branched N-glycosylation of E-Cadherin to block epithelial-mesenchymal transition and lymphatic metastasis
Source: Br J Cancer. 2020 Jul 28;123(8):1315–25. doi: 10.1038/s41416-020-1007-3 (PMC7555552; doi:10.1038/s41416-020-1007-3)
Supplement: Supplementary file 1 — Supplemental materials [file 41416_2020_1007_MOESM1_ESM.pdf]

Supplementary Materials for

Suppression of G6PD induces the expression and bisecting GlcNAc-branched N-glycosylation of E-cadherin to block epithelial-mesenchymal transition and lymphatic metastasis

Yifei Wang, Qingxiang Li, Lixuan, Niu, Le Xu, Yuxing Guo, Lin Wang*, Chuanbin Guo*

*Corresponding author.

Email: Lin Wang: linwang911@126.com

Chuanbin Guo: guodazuo@sina.com

This document includes:

Supplemental materials and methods

Supplemental table 1-4

Supplemental figure 1-7

Supplemental materials and methods

G6PD activity detection

G6PD activity detection was performed according to the manufacturer's instructions (Abcam, Cambridge, MA, USA). Briefly, the cells were seeded in a 6-well plate at a density of 4×10^5 cells per well. After treated with DHEA, total proteins were extracted from the cells using RIPA buffer (Huaxingbio, Beijing, China) containing a protease inhibitor. Protein concentration was measured by BCA method, and all samples were equalled to same concentration. 50 μL of standard dilutions (prepared with NADH standard) and samples were added into a 96-well plate. Each sample was added to four wells with three detection wells and one background well. Then, 50 μL of G6PD reaction mix was added into each standard and sample wells, and 50 μL of G6PD background mix was added into the background wells. The OD value was and measured at 450 nm on a microplate reader 10 min and 20 min later. The amount of NADH in sample well calculated from standard curve, and G6PD activity was calculated by dividing NADH amount with reaction time (10 min).

Immunoprecipitation of E-Cadherin

After treatment by siRNA, total protein was extracted from the cells following protein extraction protocol described in Western blot. E-Cadherin in each 100 μl of protein lysate was then immunoprecipitated by adding 1 μl of anti-E-Cadherin monoclonal antibodies (CST) and 15 μl of a Protein G Agarose Suspension (Millipore), followed by an overnight rotation at 4°C. Thereafter, the antibody-antigen-agarose complexes were separated by centrifuge, and washed by protein extracting solution for three times. 3×loading buffer (with SDS) was used to break the complex at 100°C for 15min.

N-glycan structures analysis by mass spectrometry

Immunoprecipitated E-Cadherin were suspended in a reduced sample buffer, heated to 100°C for 10min, resolved by 10% (w/v) SDS-PAGE, and the gel was excised and cut into pieces. The gel pieces were distained and dehydrated with acetonitrile. Then the protein in gel was reduced and alkylated by the incubation with dithiothreitol and iodoacetamide. The protein was cut by trypsin at the concentration of 15 ng/μL, and the peptides were extracted by extraction solution (5% trifluoroacetic acid and 50% acetonitrile were diluted in water). LC/multistage MS (MS^n) was carried out on an Elite Hybrid Ion Trap-Orbitrap Mass Spectrometer (Thermo Fisher Scientific, USA). The eluents were 0.1% acetate, 2% acetonitrile (phase A) and 0.1% acetate, 80% acetonitrile (phase B). The peptides were separated on a capillary high-performance liquid chromatography (Thermo Fisher Scientific, USA) with a linear gradient of 6–95% phase B in 60 min. A full MS^1 scan (m/z 350–1550) followed by data dependent MS^2 for the most abundant ions was performed in HCD modes. The data were analysed by Byonic Software (Protein Metrics).

Metabolites associated with PPP analysis by mass spectrometry

After treated by siRNA, the cells were digested by 0.25% trypsin, followed by three-time PBS washing. Liquid Chromatography was performed by means of a UHPLC system (Shimadzu, Kyoto, Japan) equipped a LC-30AD solvent delivery system, a SIL-30AC autosampler, a CTO-30A column oven, a DGU-20A3 degasser and a CBM-20A controller. The separation of the compounds was carried out on Agilent XDB C18 (3.5 um, 3.0 X 100 mm) operated at 40°C. The eluents were 10 mM Dihexylammonium Acetate (DHAA) in water, pH=5.0 (phase A) and 10 mM DHAA in acetonitrile (phase B). They were delivered at a flow rate of 0.5 mL/min under a gradient program. The gradient system was 0–1.0min, 2% B; 1.0–8.4 min, 2–60% B; 8.4–8.5 min, 60–95% B; 8.5–12.0 min, 95-2% B. The mass spectra were acquired using a TripleTOF™ 5500 system with a Duo Spray source (SCIEX, CA, USA) in negative ESI mosde. The data were analysed by Peak View Software 2.2 (SCIEX).

ROS detection

For the measurement of ROS, we harvested the cells treated with siRNA for 48 h and then washed the treated cells twice with PBS. Then, 1×10^6 cells were suspended in 1 mL PBS and incubated with 10 μM DCFH-DA (Sigma, St. Louis, MO, USA), a cell membrane permeable fluorescence probe. Next, the cells were washed and suspended in 1 mL of PBS for measurements. Fluorescence levels were measured using flow cytometry (Beckman CytoFLEX) with excitation and emission wavelengths set at 480 nm and 530 nm, respectively.

Construction of luciferase reporter plasmids

The pGL3-basic plasmid (Promega) was used as the backbone. The sequence of the 2000 bp ahead of the transcriptional start site of *MGAT3* was searched in the NCBI database (Gene ID: 4248, Transcript ID: NM_002409.5). The sequence was synthesised by Shanghai Shenggong Co., Ltd. in the pUC57 backbone. Kpn I and Nhe I (New England Biolabs, UK) were used for re-establishing the pGL3-MGAT3-promoter plasmid. The Fast Site-Directed Mutagenesis Kit (Tiangen biotech, Beijing, CN) was used for point mutation on the plasmid. The mutation scheme is shown in **Supplemental Figure S5B**. All the plasmids were amplified in DH5α competent cells (Tiangen biotech). The EndoFree Mini Plasmid Kit II (Tiangen biotech) was used for plasmid extraction.

Immunofluorescence and lectin fluorescence

The tissue sections were prepared as described above. For immunofluorescence, antigen retrieval and endogenous peroxidase activity blockage were performed in the same manner as used for IHC. The tissue sections were then incubated with rabbit anti-human E-cadherin antibody (1:200, CST) overnight at 4°C, followed by a FITC-labelled secondary antibody (Zhongshan Biosciences Inc.). For lectin fluorescence, the

tissue sections were blocked with 1× Carbo Free Blocking Solution (Vector Laboratories) after deparaffinisation and rehydration and were incubated with Biotinylated Phaseolus Vulgaris Erythroagglutinin (PHA-E, 1:200, Vector Laboratories) overnight at 4℃. This was followed by staining with DyLight 594 Streptavidin (Vector Laboratories) for 1 h at RT. Nuclear staining was performed by incubation with DAPI (Zhongshan Biosciences Inc.). The images were then scanned with an optimal microscope (Olympus).

Supplemental Table 1 The information of primary antibodies used in Western blot

Name of antibody	Dilution rate	Brand
Rabbit anti-G6PD polyclonal antibody	1:1000	Abcam, USA
Rabbit anti-RPS18 polyclonal antibody	1:500	Abcam, USA
Rabbit anti-E-Cadherin monoclonal antibody	1:1000	CST, USA
Rabbit anti-N-Cadherin polyclonal antibody	1:1000	Absin, CN
Rabbit anti-p-JNK (Thr183/Tyr185) monoclonal antibody	1:500	CST, USA
Rabbit anti-JNK monoclonal antibody	1:1000	CST, USA
Rabbit anti-SNAIL polyclonal antibody	1:1000	Absin, CN
Rabbit anti-SLUG polyclonal antibody	1:1000	Absin, CN
Rabbit anti-TWIST1 polyclonal antibody	1:1000	Absin, CN
Rabbit anti-ZEB1 polyclonal antibody	1:1000	Absin, CN
Rabbit anti-p-AKT (Ser473) monoclonal antibody	1:500	CST, USA
Rabbit anti-AKT (pan) monoclonal antibody	1:1000	CST, USA
Rabbit anti-p-GSK-3β (Ser9) monoclonal antibody	1:500	CST, USA
Rabbit anti-GSK-3β monoclonal antibody	1:1000	CST, USA
Rabbit anti-MGAT3 (N-terminal) polyclonal antibody	1:400	Abcam, USA
Rabbit anti-p-c-Jun (Ser73) monoclonal antibody	1:500	CST, USA
Rabbit anti-c-Jun monoclonal antibody	1:1000	CST, USA

Supplemental Table 2 The sequence of the primers used in qPCR

Name of gene	Forward primer (5'→3')	Reverse primer (5'→3')
MGAT3	CCCACTCTACTCCCACTCG	CGCACGAAATACTCGGTGGT
MGAT5	GCACCGGAACAAACTCAACC	AAGAGGGCCACCACTGAAAG
RPS18	GCGGCGGAAAATAGCCTTTG	GATCACACGTTCCACCTCATC
MGAT3-P1 (ChIP)	TCCGCCTCCTGGGTTCAAGC	TTAGCCGGGTGTGGTGGCAC
MGAT3-P2(ChIP)	GGTTTCATCATGTTGTCAGGCT	GGTTTCATCATGTTGTCAGGCT

Supplemental Table 3 The relationship between G6PD expression and clinical characters of OSCC

	Total	G6PD expression		*P* value
		Low	High	
**Total**	105	60	45	
**Age**				
≤ 55	43	22	21	0.302
> 55	62	38	24	
**Gender**				
Male	72	38	34	0.182
Female	33	22	11	
**Growth pattern**				
Exophytic	37	19	18	0.510
Ulcerative	39	22	17	
Infiltrating	29	19	10	
**Pathological grading**				
I	45	32	13	0.012
II-III	60	28	32	
**Clinical stage**				
I-II	41	29	12	0.024
III-IV	64	31	33	
**T stage**				
1-2	66	43	23	0.031
3-4	39	17	22	
**Lymphatic metastasis**				
No	55	41	14	<0.0001
Yes	50	19	31	

Supplemental Table 4 The information of complex N-glycans in WSU-HN6 cell line detected by mass spectrometry

	Deduced type	Peptide	Glycans NHFAGNa	Observed m/z	Scan Time	Intensity
siNC	Bisecting	R.TIFFC[+57.021]ERNPKPQVINIIDADLPPN[+2481.889]TSPFTAELTHGASANWTIQYNDPT.Q	HexNAc(6)Hex(6)NeuAc(1)	330.041	56.033	13832000
	Bisecting	R.TIFFC[+57.021]ERNPKPQVINIIDADLPPN[+3067.115]TSPFTAELTHGASANWTIQYND.P	HexNAc(8)Hex(8)Fuc(1)	346.025	56.366	7525600
	β1,6-	L.LVFDYEGSGSEAASLSSLN[+1663.608]SSESDKDQDYDYLNEWGNR.F	HexNAc(5)Hex(4)	312.039	56.771	5991700
	β1,6-	L.SSLN[+2717.978]SSESDK.D	HexNAc(7)Hex(8)	539.641	23.509	4513600
siG6PD	Bisecting	K.AADTDPTAPPYDSLLVFDYEGSGSEAASLSSLN[+2158.803]SSESDKDQDYDYLNEWGNRFK.K	HexNAc(6)Hex(4)Fuc(2)	353.023	53.835	737620
	Bisecting	E.GSGSEAASLSSLN[+2028.740]SSESDKDQDYDYLNEWGNR.F	HexNAc(6)Hex(5)	307.025	55.191	1719800
	Bisecting	R.EDFEHVKNSTYTALIIATDN[+1907.714]GSPVATGTGTLLLILSDVNDNAPIPEPRTIFFC[+57.021]ER.N	HexNAc(7)Hex(3)	468.011	22.217	53761000
	Bisecting	Y.DSLLVFDYEGSGSEAASLSSLN[+2053.772]SSESDKDQDYDYLNEWGNRFK.K	HexNAc(7)Hex(3)Fuc(1)	329.134	56.322	13832000
	β1,6-	R.TIFFC[+57.021]ERNPKPQVINIIDADLPPNTSPFTAELTHGASAN[+2432.884]WTIQYNDPTQESIILKPKMALEVGDYK.I	HexNAc(6)Hex(3)Fuc(1)NeuAc(2)	619.02	30.629	5143600
	β1,6-	Y.LPRPANPDEIGNFIDENLKAADTDPTAPPYDSLLVFDYEGSGSEAASLSSLN[+1866.688]SSESDKDQDYDYLNEWGNR.F	HexNAc(6)Hex(4)	323.018	56.558	1900000
	β1,6-	R.TIFFC[+57.021]ERNPKPQVINIIDADLPPNTSPFTAELTHGASAN[+2465.894]WTIQYND.P	HexNAc(6)Hex(5)Fuc(1)NeuAc(1)	385.021	20.713	2109400
	β1,6-	T.APPYDSLLVFDYEGSGSEAASLSSLN[+4028.418]SSESDKDQDYDYLNEWGNR.F	HexNAc(7)Hex(8)Fuc(1)NeuAc(4)	334.964	56.707	1629700

Supplemental figures

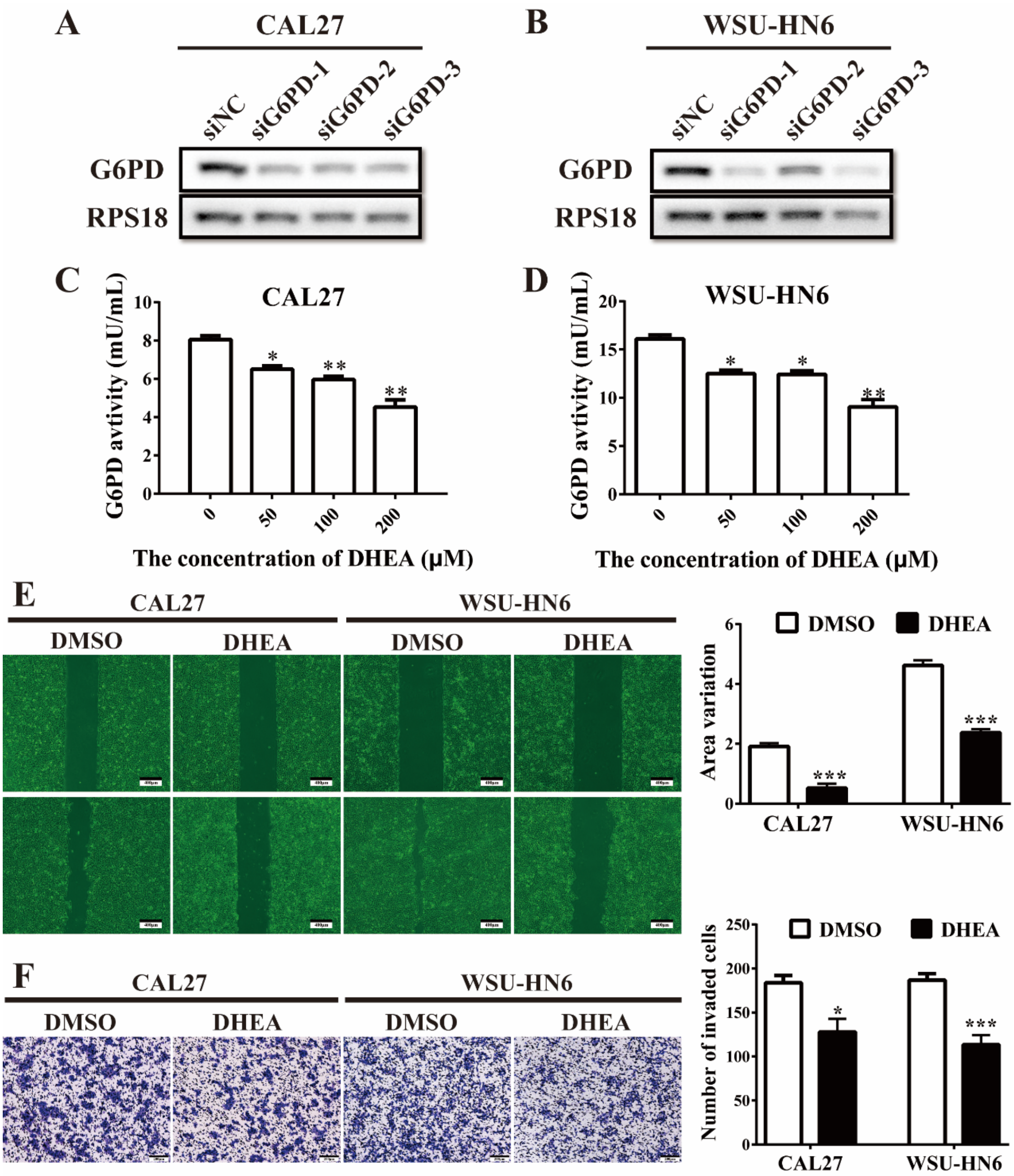

Supplemental Figure 1. (A-B) Western blot analysis of G6PD in CAL27 **(A)** and WSU-HN6 **(B)** cells treated with siNC or siG6PD for 72 h. Densitometric analysis of protein expression relative to RPS18 levels. **(C-D)** G6PD activity analysis of CAL27 **(C)** and WSU-HN6 **(D)** cells treated with DMSO or DHEA (50 μM, 100 μM, or 200 μM) (n=3 per group). **(E)** Wound healing assays in CAL27 and WSU-HN6 cells treated with DMSO or DHEA (50 μM). Five different fields were recorded and measured. **(F)** Transwell assays in CAL27 and WSU-HN6 cells treated with DMSO or DHEA (50 μM). Five different fields were recorded and measured. The data in the graph are presented as the mean ± SEM. *: P<0.05, **: P<0.01.

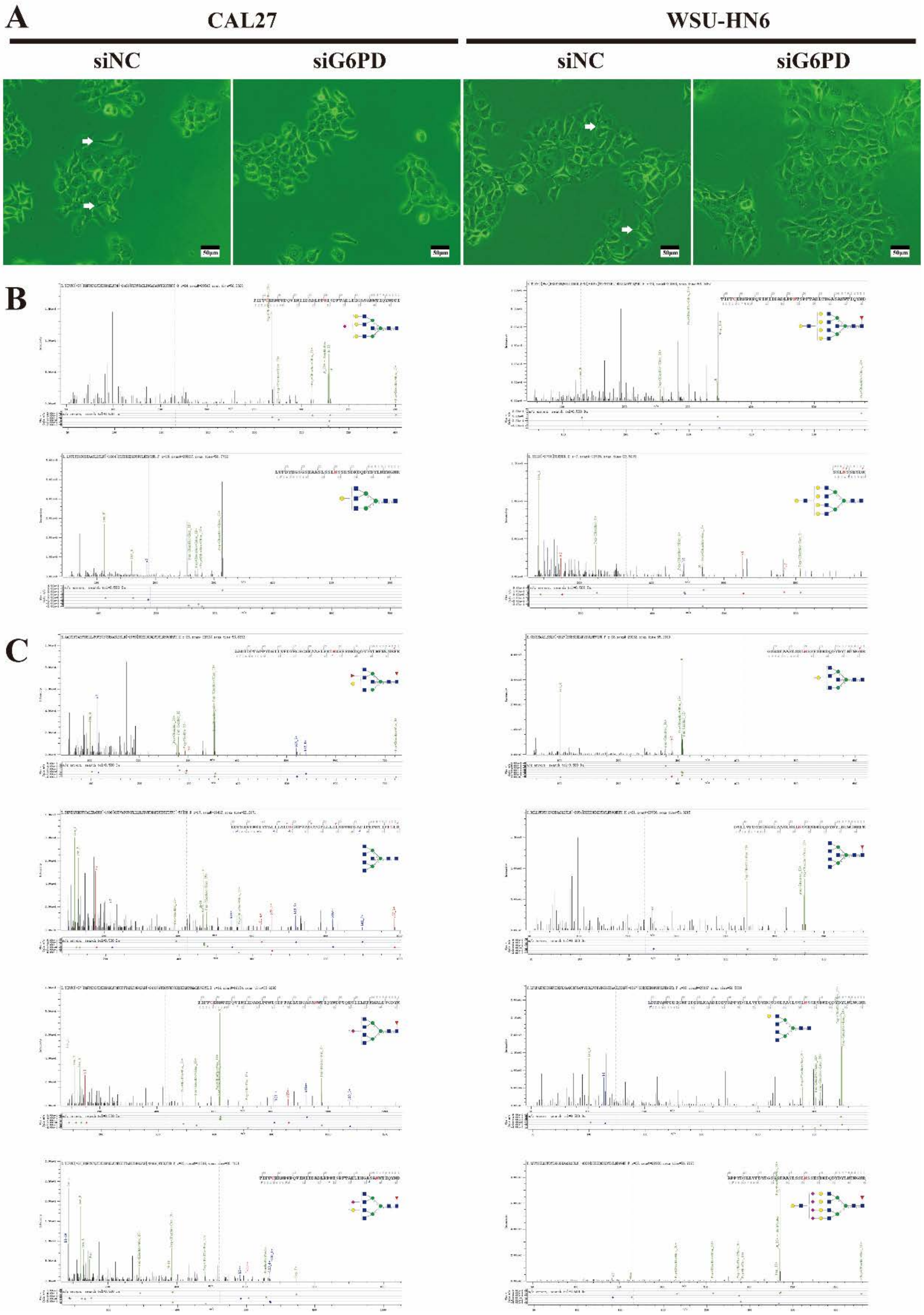

Supplemental Figure 2. (A) The cell patterns of CAL27 and WSU-HN6 cell lines after treatment with siNC or siG6PD. White arrows indicate abnormal branches. **(B-C)** The MS2 spectrum and deduced structures of N-glycans in WSU-HN6 cell lines treated with siNC **(B)** or siG6PD **(C)**. Bisecting GlcNAc-branced N-glycans was deduced based on the presence of "Pep+3HexNAc+3Hex" in MS^2 spectrum. ▼: fucose, ●: galactose, ●: mannose, ■: N-acetylglucosamine, ◆: sialic acid (NeuAc).

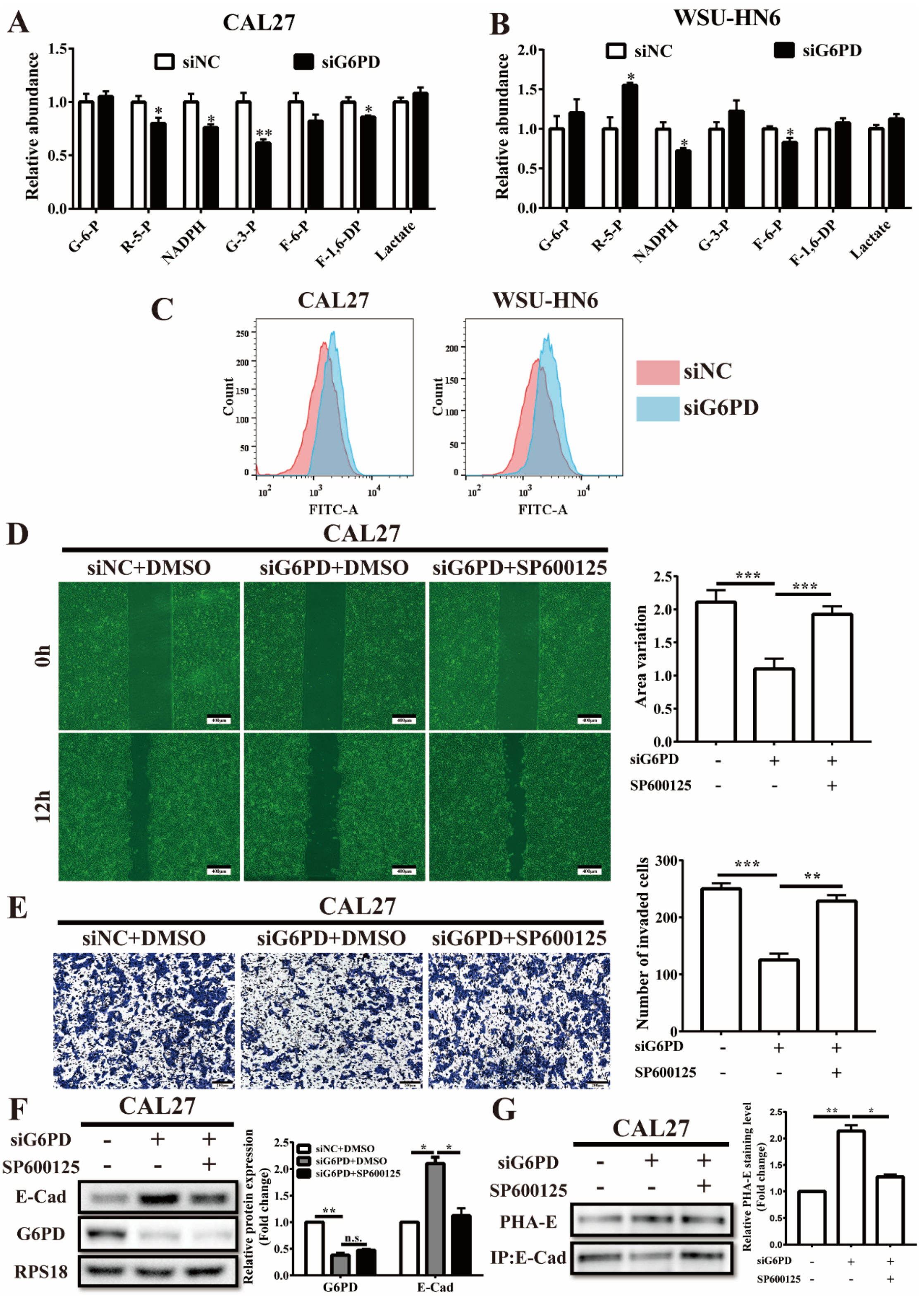

Supplemental Figure 3. (A-B) Metabolomics analysis of metabolites associated with PPP in CAL27 **(A)** and WSU-HN6 **(B)** cell lines (n=5 per group). G-6-P: glucose-6-phosphate, R-5-P: ribose-5-phosphate, G-3-P: glyceraldehyde-3-phosphate, F-6-P: fructose 6-phosphate, F-1,6-DP: fructose-1,6-diphosphate. **(C)** Flow cytometry analysis of DCFH-DA to measure ROS levels in CAL27 and WSU-HN6 cells treated with siNC or siG6PD. Red lines indicate siNC, while blue lines indicate siG6PD. **(D)**

Wound healing assays in CAL27 cells treated with siNC, siG6PD, or siG6PD with SP600125 (10 μM). Five different fields were recorded and measured. **(E)** Transwell assays in CAL27 cells treated with siNC, siG6PD, or siG6PD with SP600125 (10 μM). Five different fields were recorded and measured. **(F)** Western blot analysis of E-cadherin and G6PD in CAL27 cells treated with siNC, siG6PD, or siG6PD with SP600125 (10 μM). Densitometric analysis of protein expression relative to RPS18 levels (n=2 per group). **(G)** Lectin blot analysis of PHA-E in CAL27 cells treated with siNC, siG6PD, or siG6PD with SP600125 (10 μM). Densitometric analysis of protein expression relative to E-cadherin levels (n=2 per group). The data in the graph are presented as the mean ± SEM. *: $P<0.05$, **: $P<0.01$, ***: $P<0.001$.

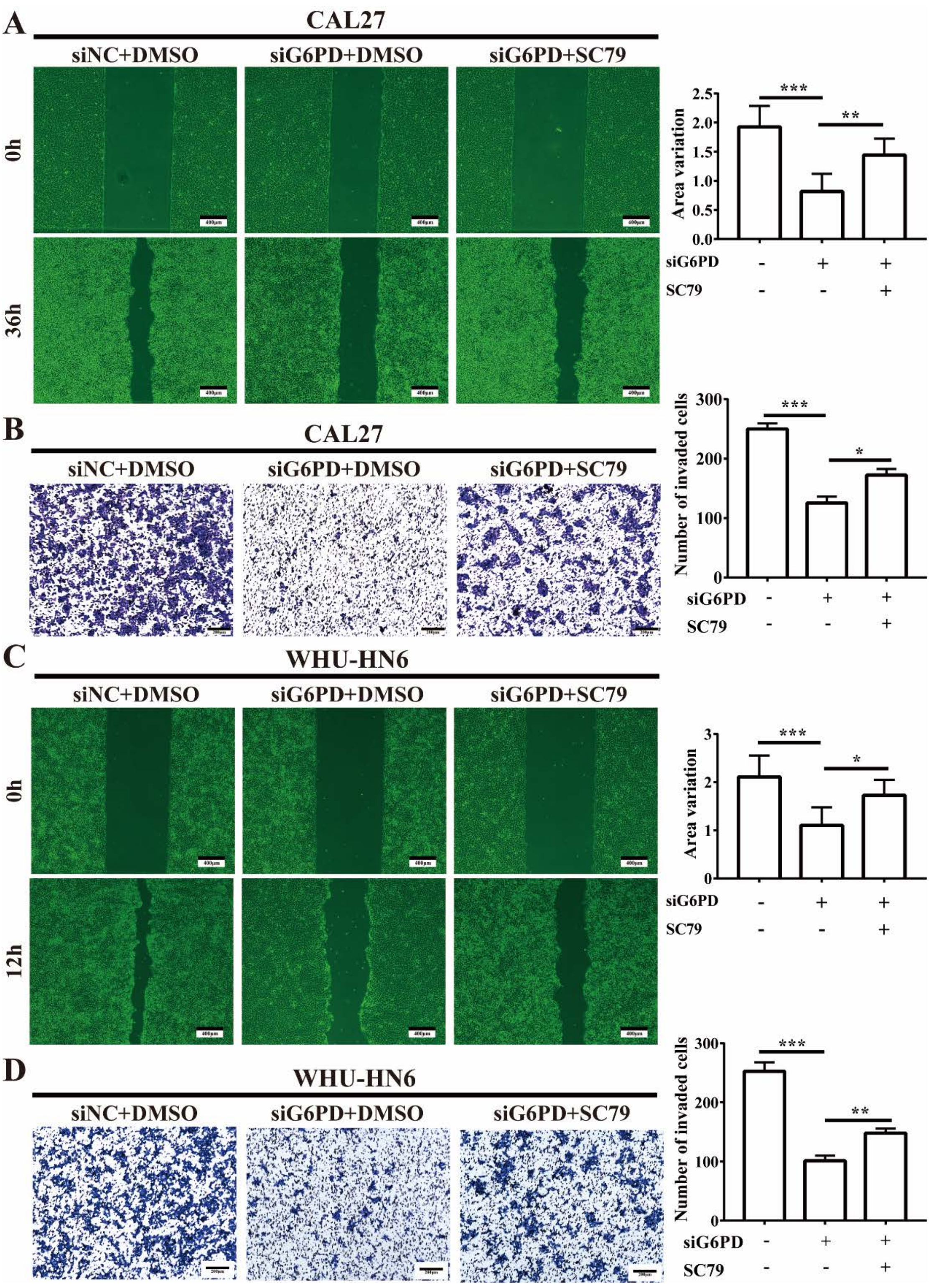

Supplemental Figure 4. (A) Wound healing assays in CAL27 cells treated with siNC, siG6PD, or siG6PD with SC79 (8 μM). **(B)** Transwell assays in CAL27 cells treated with siNC, siG6PD, or siG6PD with SC79 (8 μM). **(C)** Wound healing assays in WSU-HN6 cells treated with siNC, siG6PD, or siG6PD with SC79 (8 μM). **(D)** Transwell assays in WSU-HN6 cells treated with siNC, siG6PD, or siG6PD with SC79 (8 μM). Five different fields were recorded and measured for each assay. The data in the graph are presented as the mean ± SEM. *: P<0.05, **: P<0.01, ***: P<0.001.

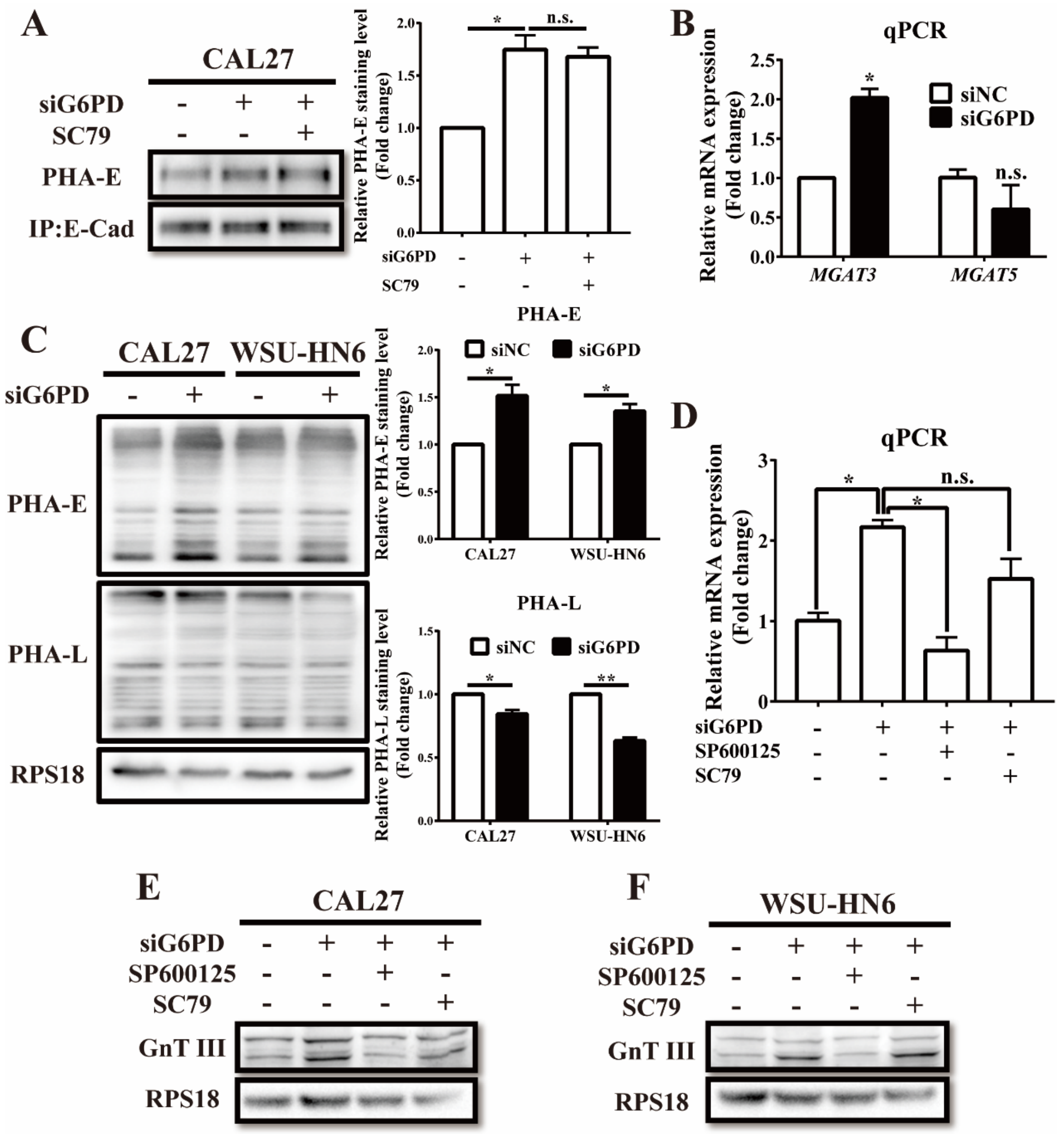

Supplemental Figure 5. (A) Lectin blot analysis of PHA-E in CAL27 cells treated with siNC, siG6PD, or siG6PD with SC79 (8 μM). Densitometric analysis of protein expression relative to E-cadherin levels (n=2 per group). **(B)** qPCR of *MGAT3, MGAT5*, and *FUT8* in CAL27 cells treated with siNC, siG6PD. mRNA expression relative to RPS18 levels (n=2 per group). **(C)** Lectin blot of total bisecting GlcNAc and β1,6-GlcNAc of whole protein lysis of CAL27 and WSU-HN6 cell lines treated with siNC or siG6PD. **(D)** qPCR of *MGAT3* in CAL27 cells treated with siNC, siG6PD, siG6PD with SP600125 (10 μM), or siG6PD with SC79 (8 μM). mRNA expression relative to RPS18 levels (n=2 per group). **(E-F)** Western blot analysis of GnT III in CAL27 **(E)** and WSU-HN6 **(F)** cells treated with siNC, siG6PD, siG6PD with SP600125 (10 μM), or siG6PD with SC79 (8 μM). Densitometric analysis of protein phosphorylation relative to RPS18 levels. The data in the graph are presented as the mean ± SEM. *: *P*<0.05, **: *P*<0.01.

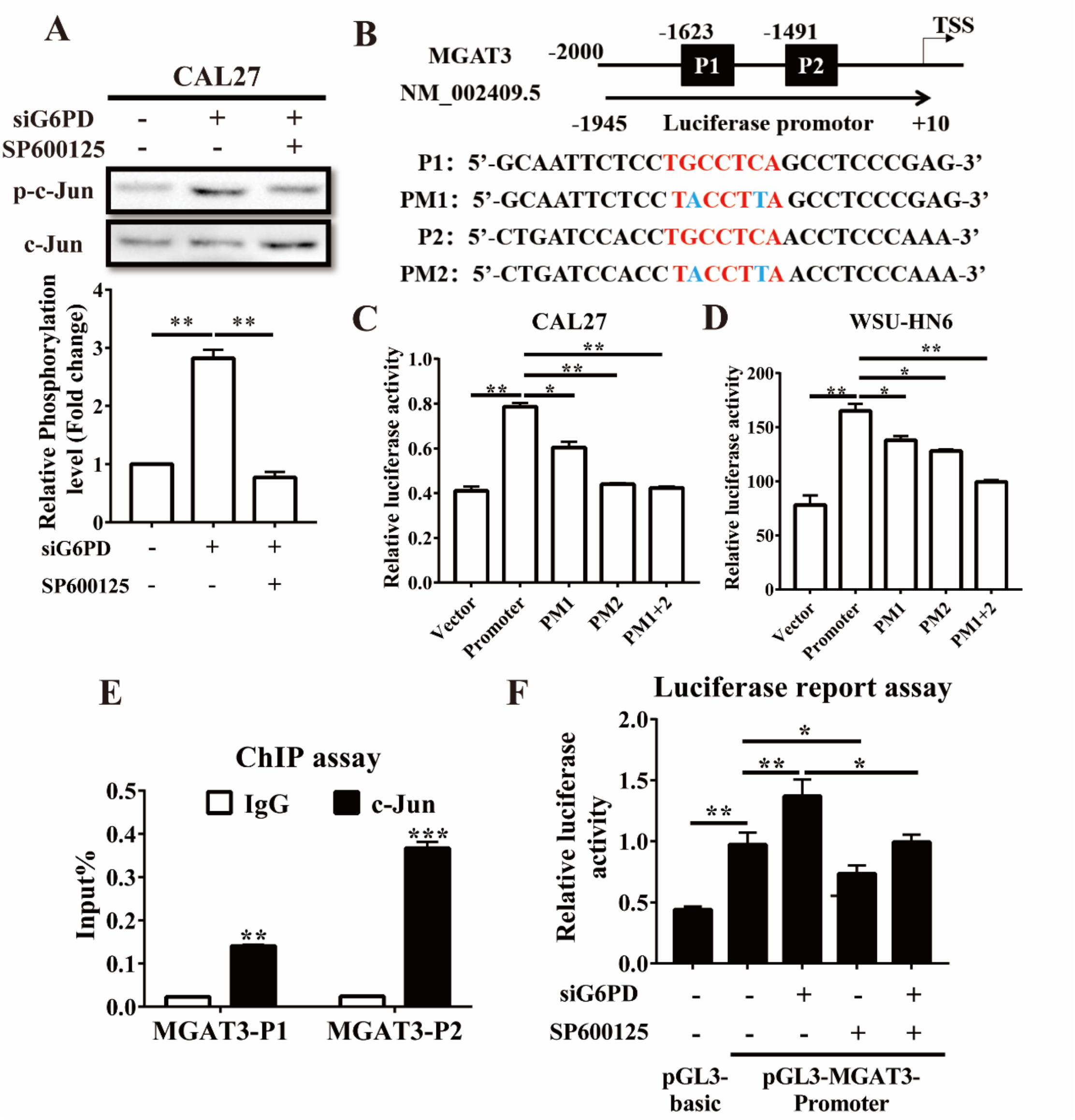

Supplemental Figure 6. (A) Western blot analysis of p-c-Jun (Ser73) in CAL27 cells treated with siNC, siG6PD, or siG6PD with SP600125 (10 µM). Densitometric analysis of protein phosphorylation relative to c-Jun levels (n=2 per group). **(B)** The scheme of the luciferase reporter plasmid and point mutation (blue characters) on the putative c-Jun binding site (red characters) of *MGAT3* promoter. **(C-D)** Luciferase reporter assay of *MGAT3* promoter function in CAL27 **(C)** and WSU-HN6 **(D)** cells with or without a point mutation on the *MGAT3* promoter (n=3 per group). **(E)** ChIP assay of c-Jun and the *MGAT3* promoter in the CAL27 cell line (n=3 per group). **(F)** Luciferase reporter assay of *MGAT3* promoter function in CAL27 cells treated with siNC, siG6PD, SP600125 (10 µM), or siG6PD with SP600125 (10 µM) (n=3 per group). The data in the graph are presented as the mean ± SEM. *: P<0.05, **: P<0.01.

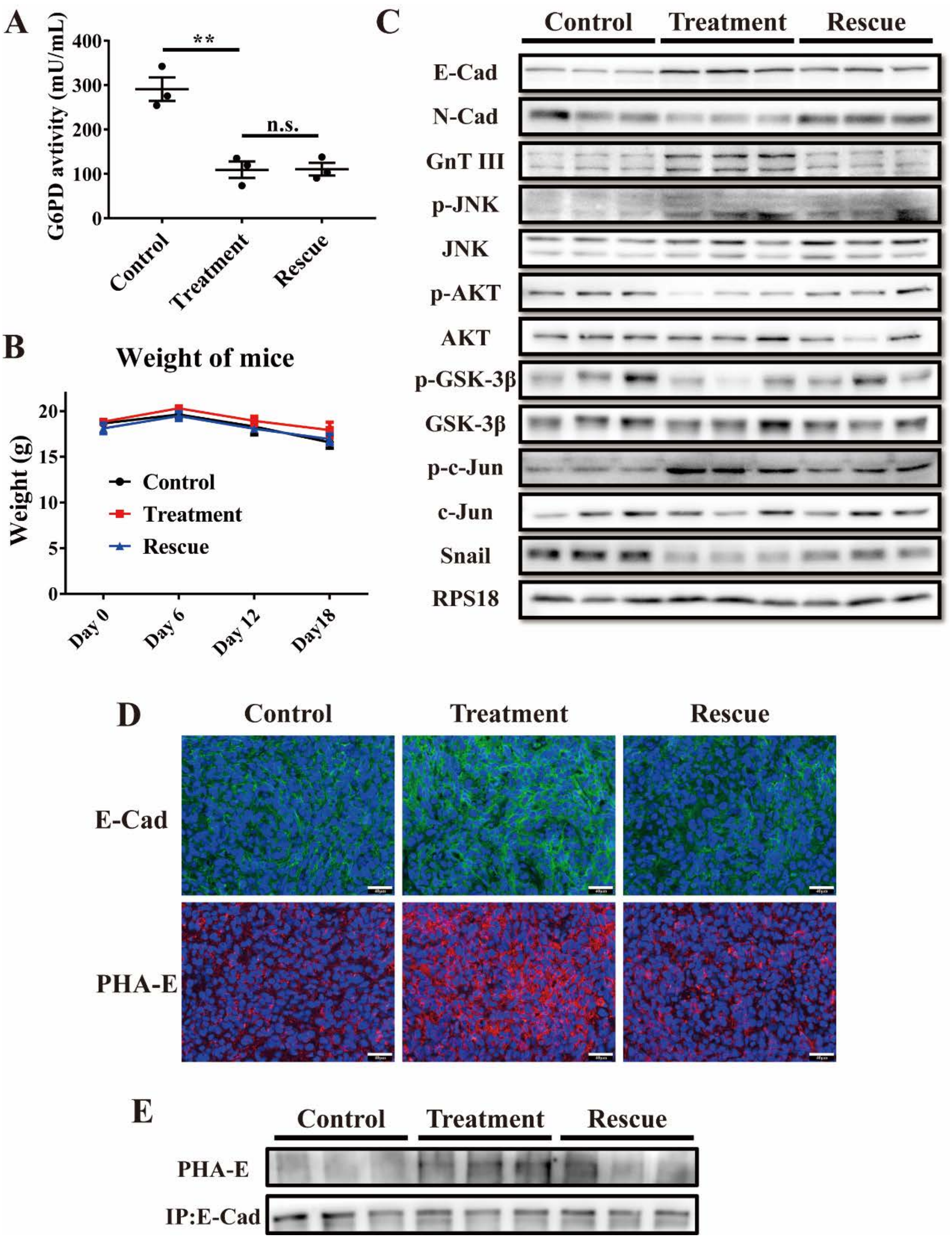

Supplemental Figure 7. (A) G6PD activity analysis in the tumour tissues of each group. **(B)** The weight change of the mice during the study. **(C)** The bands of the western blot shown in **Figure 6C-J**. **(D)** Immunofluorescence staining of E-cadherin and PHA-E in the tumour tissues of each group. **(E)** The bands of the lectin blot and western blot shown in **Figure 6K**. The data in the graph are presented as the mean ± SEM **: *P*<0.01.
